# Imaging tumour response: beyond RECIST

**DOI:** 10.1186/1470-7330-14-S1-O36

**Published:** 2014-10-09

**Authors:** Dow-Mu Koh

**Affiliations:** 1 Royal Marsden Hospital, Sutton, UK

## 

One of the most widely employed quantitative measurements for assessing tumour response to treatment is the tumour diameter (RECIST criteria), usually determined on cross-sectional CT or MRI. This simple measurement is relatively reproducible and reduction in the maximum tumour diameter by 30% or more following therapy is taken as a sign of effective treatment. However, new targeted therapies may be effective without significantly reducing tumour size and other quantitative response criteria are being evaluated.

Some of the response criteria being evaluated combine tumour diameter measurement with other quantitative assessment (e.g. CT density). These have been applied towards the evaluation of gastrointestinal stromal tumours (CHOI criteria) and renal cell carcinoma (modified or revised CHOI criteria) undergoing multi-kinase inhibitor treatment; and hepatocellular carcinoma (modified RECIST criteria) treated with novel therapeutics or chemoembolization. In these clinical settings, the applications of such criteria for tumour response assessment have shown better correlation with clinical outcome compared with conventional RECIST criteria. When immunotherapy is administered, conventional RECIST criteria may erroneously ascribe the appearance of new lesions to disease progression; whereas this phenomenon is given due consideration within the immune-related response criteria (irRC).

As quantitative imaging becomes increasingly important in oncology, quantitative indices are being applied and developed in CT, MRI and PET imaging for tumour response assessment. Using perfusion CT technique, the change in CT attenuation value with time resulting from the passage of contrast through tissue can be used to calculate quantitative vascular parameters such as permeability surface area product (PS), blood flow (F) and mean transit time (MTT). Using dual energy CT, it is possible to obtain quantitative iodine contrast distribution in soft tissue.

MRI is a powerful multiplex imaging technique because depending on how the scans are performed, it can yield different quantitative information. Two of the most widely employed quantitative techniques for tumour assessment are dynamic contrast enhanced MRI (DCE-MRI) and diffusion-weighted MRI (DW-MRI). The former has been used in early phase trials for the assessment of antivascular/ antiangiogenic therapies. The latter has shown potential as an early response biomarker to a range of effective treatments including chemotherapy, radiotherapy, chemoemobolization treatment and novel therapeutics. Other clinical MR techniques include intrinsic susceptible contrast imaging and magnetic resonance spectroscopy (MRS). However, these have currently limited value for assessing tumour response to treatment.

PET imaging using 18FDG tracer enables the semi-quantitative standardised uptake value (SUV) to be derived. PET imaging is increasing utilized for tumour response assessment, and forms the basis of standard criteria for assessment of tumour response in lymphoma. By performing dynamic imaging, it is also possible to calculate the quantitative tracer uptake in tissues. The strength of PET imaging is the range of radiolabelled tracers that are now available clinically to probe a range of tissue properties.

In early phase trials, quantitative imaging other than tumour size measurements using CT, MRI or PET can provide mechanistic information about drug action, and also therapeutic effects on specific aspects of tumour biology (e.g. vascularity, cellularity). By using multimodality imaging, there is also an opportunity to corroborate imaging measurements made using one technique with another. However, quantitative techniques require a process of quality control and quality assurance; as well as knowledge of their measurement reproducibility so that they can be applied with confidence in clinical practice.

